# Moderately Hypofractionated Online Adaptive Radiotherapy for Cervical Cancer: A Prospective Study of Feasibility, Acute Toxicity and Dosimetric Benefits

**DOI:** 10.3390/cancers18152493

**Published:** 2026-08-04

**Authors:** Zheng Zeng, Yining Chen, Xiangyin Meng, Yuliang Sun, Junfang Yan, Ke Hu, Fuquan Zhang

**Affiliations:** 1 Department of Radiation Oncology, Peking Union Medical College Hospital, Chinese Academy of Medical Sciences & Peking Union Medical College, No. 1 Shuaifuyuan Wangfujing, Dongcheng District, Beijing 100730, China; zengzheng1206@163.com (Z.Z.); mengxiangyin@pumch.cn (X.M.); syl10710@163.com (Y.S.); 2 Eight-Year Medical Doctor Program, Chinese Academy of Medical Sciences & Peking Union Medical College, Beijing 100730, China; chenyn19@student.pumc.edu.cn; 3 Department of Radiation Oncology, State Key Laboratory of Complex Severe and Rare Diseases, Peking Union Medical College Hospital, Chinese Academy of Medical Sciences & Peking Union Medical College, No. 1 Shuaifuyuan Wangfujing, Dongcheng District, Beijing 100730, China

**Keywords:** cervical cancer, moderately hypofractionated radiotherapy, online adaptive radiotherapy, feasibility, acute toxicity, dosimetric analysis

## Abstract

Background/Objectives: Moderately hypofractionated radiotherapy (MHRT) may shorten treatment duration for cervical cancer but raises concerns regarding toxicity due to substantial interfractional pelvic organ motion. This prospective study evaluated the feasibility, workflow efficiency, dosimetric benefits, and acute toxicities of daily online adaptive radiotherapy (oART)-guided MHRT. Methods: Thirty patients with FIGO 2018 stage IB1–IIB or IIIC1 cervical squamous cell carcinoma receiving definitive chemoradiotherapy were prospectively included between September 2023 and April 2024 (NCT05994300). All patients underwent daily oART, receiving 43.35 Gy in 17 fractions to the pelvic target volume, with a simultaneous integrated boost to 54.40 Gy in 17 fractions for involved lymph nodes. The adaptive workflow consisted of iterative cone-beam computed tomography acquisition, artificial intelligence-assisted contouring, physician review, plan adaptation, and treatment verification. Workflow efficiency, plan selection, target coverage, organ-at-risk (OAR) sparing, treatment completion, early tumor response and acute toxicity were prospectively assessed. Results: A total of 510 adaptive fractions were delivered. The first-attempt adaptation success rate was 99.0%, and adapted plans were selected in 99.4% of fractions. The mean adaptive workflow and total treatment times were 17.3 and 23.3 min per fraction, respectively. Compared with scheduled plans, adapted plans significantly improved target coverage, with V_100%_ increasing by 7.26% for the planning target volume of the uterus and 8.79% for planning target volume of the cervix (both *p* < 0.001), while significantly reducing doses to the bladder, rectum, small bowel, bone marrow, and femoral heads. All patients achieved complete clinical response at 3 months. Acute toxicity was generally manageable; Grade ≥ 3 gastrointestinal, genitourinary, and hematologic toxicities occurred in 10%, 0%, and 40% of patients, respectively, including one Grade 4 neutropenia event, and no treatment interruptions. Conclusions: Daily oART-guided MHRT was feasible and efficient, providing improved target coverage and reduced OAR doses compared with scheduled plans. Acute toxicity was acceptable. These findings provide early prospective evidence supporting the feasibility of this treatment strategy and warrant further validation in larger prospective studies with longer follow-up.

## Simple Summary

Radiotherapy is a key treatment for cervical cancer, but conventional treatment schedules are lengthy and place a substantial burden on both patients and healthcare systems. Shorter radiotherapy schedules may improve efficiency; however, concerns about toxicity have limited their adoption because pelvic organs can move considerably between treatment sessions. This prospective study provides early evidence supporting the integration of daily online adaptive radiotherapy into a moderately hypofractionated definitive chemoradiotherapy regimen for cervical cancer. By adapting the treatment plan before each fraction to account for daily anatomical variations, this approach improved target coverage while reducing unnecessary irradiation of surrounding normal tissues. Among the 30 prospectively enrolled patients, treatment was completed successfully in all cases, acute toxicities were generally manageable, and early treatment responses were observed. These findings provide early prospective evidence supporting the feasibility of integrating daily online adaptive radiotherapy with moderately hypofractionated definitive chemoradiotherapy for cervical cancer and warrant further validation in larger prospective studies with longer follow-up.

## 1. Introduction

Cervical cancer is the fourth most common malignancy among women worldwide, with over 80% of cases occurring in developing countries [1,2]. External beam radiotherapy (EBRT) with brachytherapy and concurrent chemotherapy remains the standard of care for locally advanced cervical cancer [3]. Overall treatment duration typically requires eight weeks or longer [4,5]. However, prolonged treatment courses burden medical systems and, more importantly, are linked to poorer locoregional control and survival, underscoring the need for more efficient EBRT strategies, especially in regions with limited radiotherapy resources [6,7,8].

Over the past two decades, three-dimensional planning, intensity-modulated radiation therapy (IMRT), and image-guided techniques have represented major technological advances in radiotherapy [9]. With advances in radiotherapy and improved treatment precision, moderately hypofractionated radiotherapy (MHRT) has become a standard strategy for several common cancers, including prostate and breast malignancies [10,11]. For localized prostate cancer, MHRT is safe and effective, with regimens such as 60 Gy/20 fractions, 70.2 Gy/26 fractions, and 70 Gy/28 fractions, reducing the treatment course from eight weeks to roughly four to six weeks [12]. MHRT shortens treatment time, improves patient compliance, and reduces medical resource use and socioeconomic burden.

Evidence for MHRT in cervical cancer, however, remains limited. In a prospective single-center randomized trial, MHRT (40 Gy/15 fractions) with weekly cisplatin and brachytherapy resulted in a significantly higher acute Grade ≥ 3 gastrointestinal toxicity rate than conventional chemoradiation (45 Gy/25 fractions; 27.6% vs. 6.7%) [13]. MHRT for cervical cancer provides efficacy comparable to conventional fractionation but may lead to higher toxicity, likely due to substantial interfractional motion of the cervix, uterus, and adjacent organs that introduces dose uncertainty [14,15]. The introduction of online adaptive radiotherapy (oART) offers a promising solution to the limitations of hypofractionation by enabling daily plan reoptimization based on real-time anatomy and reducing planning target volume (PTV) margins [16,17]. Our prior work validated an oART workflow for postoperative gynecologic cancers, demonstrating that interfractional variations can be effectively managed and clinical target volume (CTV)-to-PTV margins safely reduced to 5 mm without compromising coverage [16,18]. Prospective studies in definitive cervical cancer have similarly shown high target coverage, reduced margins, and low acute toxicity with oART [19]. Building upon our previous studies of postoperative and definitive cervical cancer oART, the present investigation extends this adaptive treatment strategy to an MHRT regimen with a predefined dose-fractionation schedule.

Previous studies have evaluated oART in postoperative gynecologic malignancies and definitive cervical cancer, mainly focusing on margin reduction, target coverage, workflow feasibility, and acute toxicity [16,19]. Separately, MHRT has been explored in cervical cancer without routine daily oART, but toxicity concerns remain because of substantial interfractional motion of the uterus, cervix, bladder, rectum, and bowel [13,14]. Therefore, the present cohort differs from previous reports by prospectively integrating definitive cervical cancer treatment, MHRT, daily cone beam computed tomography (CBCT)-guided oART, concurrent cisplatin chemotherapy, and image-guided brachytherapy within a single predefined treatment protocol. The aim of this study was not to introduce oART as a novel platform, but to provide early prospective feasibility and safety data for using daily oART to support MHRT for cervical cancer.

## 2. Materials and Methods

### 2.1. Study Population

**Figure 1:**
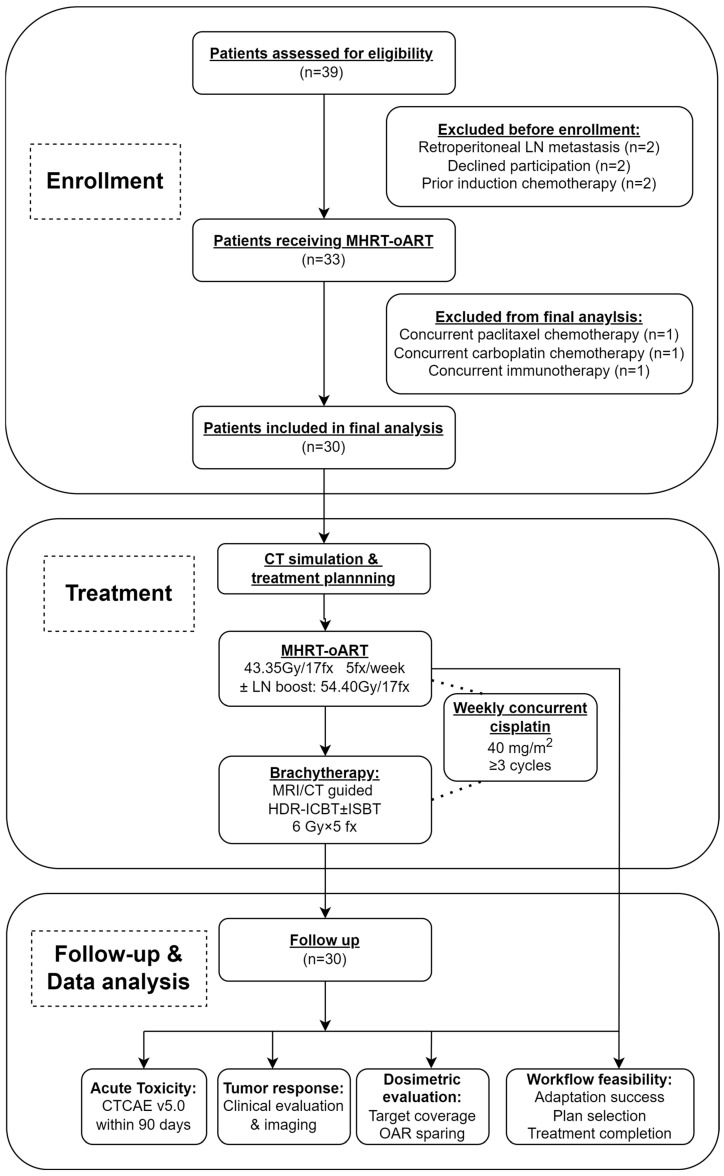
Participant flow diagram and overall study workflow. MHRT-oART, moderately hypofractionated online adaptive radiotherapy; LN, lymph node; CT, computed tomography; MRI, magnetic resonance imaging; HDR-ICBT, high-dose-rate intracavitary brachytherapy; ISBT, interstitial brachytherapy; OAR, organ at risk; Gy, gray; fx, fraction.

Thirty patients with histologically confirmed cervical squamous cell carcinoma who received definitive concurrent chemoradiotherapy were included in this prospective study between September 2023 and April 2024. Eligible patients were 18–70 years old, had International Federation of Gynecology and Obstetrics (FIGO) 2018 stage IB1–IIB or IIIC1 disease (pelvic lymph nodes ≤ 1.5 cm, ≤2 positive nodes, and no common iliac involvement), had an Eastern Cooperative Oncology Group performance status of 0–2, and were suitable for definitive radiotherapy with weekly cisplatin and brachytherapy. Key exclusion criteria included prior cervical surgery, previous abdominal or pelvic radiotherapy, pregnancy or breastfeeding, active infection, or comorbidities that could compromise protocol adherence. The patient selection process and overall study workflow are summarized in Figure 1. This study was reviewed and approved by the Institutional Review Board of Peking Union Medical College Hospital (Approval No. K4477), and all patients provided written informed consent. The trial is registered at ClinicalTrials.gov (NCT05994300).

### 2.2. Pre-Treatment Workup

Before enrollment, all patients underwent a comprehensive evaluation that included laboratory tests (complete blood count, liver and renal function tests, squamous cell carcinoma antigen, and screening for common infectious diseases), imaging examinations (pelvic magnetic resonance imaging [MRI] and chest and abdominal computed tomography [CT]), and a gynecologic physical examination. In addition, all patients underwent positron emission tomography–computed tomography (PET-CT) prior to treatment to ensure accurate tumor staging.

### 2.3. CT Simulation

All patients underwent CT simulation using the Revolution ES system (GE HealthCare, Chicago, IL, USA) in the supine position with thermoplastic immobilization and received intravenous contrast. Before scanning, patients were instructed to empty both the bladder and rectum 1 h and 40 min prior to the scheduled CT, then drink approximately 500 mL of water within 10 min. Patients were positioned in the supine position with their arms raised above the head and a vaginal marker tube was inserted to the vaginal apex. CT images were acquired with a slice thickness of 5 mm.

The scan range extended at least 5 cm beyond the superior and inferior borders of the target volume and encompassed all organs at risk (OARs) requiring evaluation. The typical coverage spanned from the upper border of L3 to 5 cm below the ischial tuberosities. For patients without contrast allergy or renal impairment, both non-contrast and contrast-enhanced CT scans were obtained for treatment simulation.

### 2.4. EBRT

**Figure 2:**
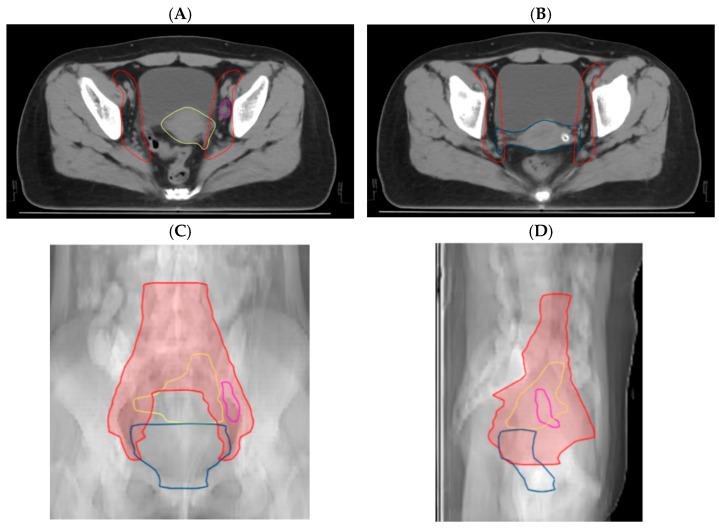
Illustration of target delineation for online adaptive radiotherapy of cervical cancer in axial (**A**,**B**), coronal (**C**), and sagittal (**D**) views. CTV-N is shown in red, CTV-U is shown in yellow, CTV-C is shown in blue, and gross tumor volume of lymph nodes is shown in magenta. CTV-N, clinical target volume of the lymph nodes; CTV-U, clinical target volume of the uterus; CTV-C, clinical target volume of the cervix.

All patients received oART using the Ethos platform (Varian Medical Systems, Palo Alto, CA, USA). In the EBRT workflow, target volumes and OARs were delineated based on Radiation Therapy Oncology Group guidelines for definitive cervical cancer radiotherapy and refined to account for interfractional anatomical changes specific to oART, incorporating our team’s clinical experience [19,20]. As shown in Figure 2, the CTV was divided into three subregions. The clinical target volume of the cervix (CTV-C) included the cervical tumor, adjacent parametrial tissues, and vaginal involvement, with boundaries extending from the posterior bladder wall anteriorly to the anterior rectal wall posteriorly, roughly covering the anterior one-third of the rectal mesentery. The clinical target volume of the uterus (CTV-U) extended from the uterine fundus to the level where the parametrium and uterosacral ligaments are visualized on the planning CT. The clinical target volume of the lymph nodes (CTV-N) encompassed the internal iliac, obturator, external iliac, presacral, and common iliac lymph node regions. Contouring was guided by findings from physical examination, MRI, and contrast-enhanced CT. PTVs were created by uniform expansions of the CTVs: 10 mm for CTV-U, 5 mm for CTV-C and CTV-N, and 3 mm for the gross tumor volume of lymph nodes [16,18]. OARs included the bladder, rectum, small bowel, femoral heads, bone marrow, and spinal cord. Bone marrow was manually contoured as the medullary cavity and cancellous bone regions within the pelvic bones, lower lumbar vertebrae, sacrum, and proximal femora on the planning CT.

The prescription dose for EBRT of cervical cancer was 43.35 Gy in 17 fractions (2.55 Gy/fraction, 1 fraction/day, 5 fractions/week). For involved lymph nodes, a simultaneous integrated boost of 54.40 Gy in 17 fractions (3.20 Gy/fraction) was applied. Assuming an α/β ratio of 10 Gy for tumor and other early-responding tissues and 3 Gy for late-responding normal tissues [21], the corresponding equivalent doses in 2-Gy fractions (EQD2) were 45.34 Gy and 59.84 Gy, respectively. At least 95% of the PTV was required to receive the full prescribed dose, while no more than 1% of the PTV was permitted to exceed 110% of the prescription. The dose constraints were based on our previously published treatment protocol [22].

The prescription regimen of 43.35 Gy in 17 fractions was selected based on both radiobiological equivalence and clinical feasibility. This schedule provides an EQD2 comparable to the conventional pelvic dose of 45 Gy in 25 fractions while avoiding the substantially larger fraction sizes used in some previous hypofractionated regimens, which have been associated with increased gastrointestinal toxicity [7,13]. The regimen was therefore designed to balance treatment acceleration with acceptable normal tissue tolerance and maintain compatibility with subsequent image-guided brachytherapy.

### 2.5. oART Workflow

**Figure 3:**
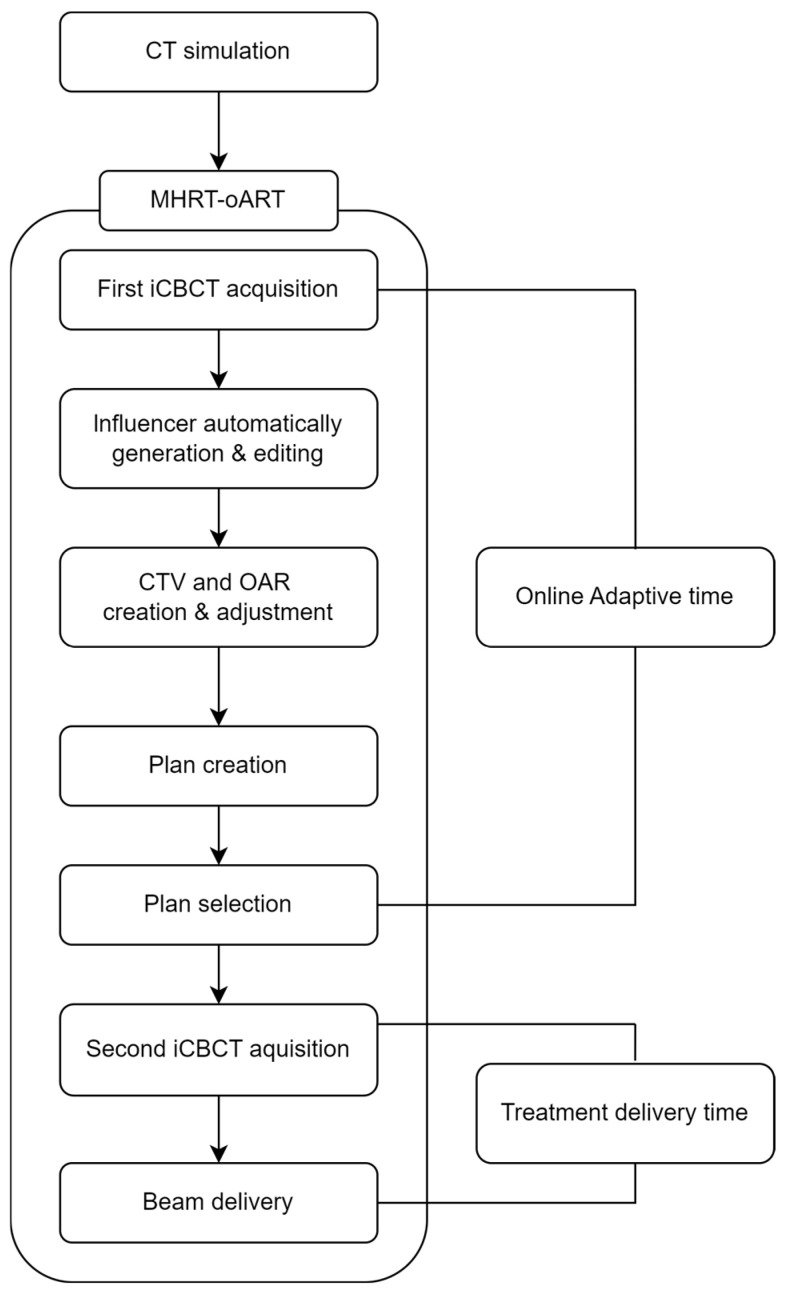
Workflow of daily online adaptive moderately hypofractionated radiotherapy for cervical cancer. CT, computed tomography; MHRT, moderately hypofractionated radiotherapy; oART, online adaptive radiotherapy; iCBCT, iterative cone-beam computed tomography; CTV, clinical target volume; OAR, organ at risk.

For each treatment fraction, the first iterative cone-beam computed tomography (iCBCT) was acquired after bladder and rectal preparation, similar to the procedure used during simulation. The automatically generated structures of the influencers (bladder, rectum, and bowel) and CTVs were reviewed and manually edited by a physician. Subsequently, both the adapted plan and the scheduled plan were generated. The scheduled plan represented the original reference plan recalculated on the daily iCBCT-derived anatomy after image registration, without reoptimization, whereas the adapted plan was newly reoptimized using the physician-reviewed target and OAR contours of the day. Both plans were evaluated on the same daily anatomy using identical target structures and planning objectives. Plan selection was based on predefined clinical planning objectives, prioritizing adequate target coverage (PTV V100% ≥ 95% and V95% ≥ 99%, whenever achievable) while minimizing OAR doses according to our institutional protocol. When both plans met the target coverage objectives, the plan providing greater OAR sparing was selected. After approval of the selected treatment plan, a second iCBCT was performed to verify the positioning of the target volumes and OARs, followed by treatment delivery. For each treatment fraction, workflow timing data, treatment completion, and dosimetric outcomes of both the adapted and scheduled plans were recorded. Figure 3 illustrates the detailed treatment workflow.

The first-attempt adaptation success rate was defined as the proportion of treatment fractions in which the adaptive workflow was successfully completed after the initial iCBCT acquisition without requiring a repeat adaptive workflow. Re-entry into the adaptive workflow was required when treatment could not proceed after the initial adaptation because of inadequate patient preparation (e.g., bladder or bowel filling), unacceptable target positioning or coverage, or clinically unacceptable plan quality resulting from substantial intrafractional anatomical changes.

### 2.6. Concurrent Chemotherapy

All patients received weekly cisplatin at a dose of 40 mg/m^2^, with dose reductions allowed for those with renal dysfunction, and a minimum of three cycles was required. Concurrent cisplatin could be administered during both EBRT and brachytherapy. Neoadjuvant or adjuvant chemotherapy was not permitted.

### 2.7. Brachytherapy

After EBRT, all patients received MRI- or CT-guided high-dose-rate intracavitary brachytherapy, with or without interstitial brachytherapy, delivering a total of 30 Gy in five fractions. According to International Commission on Radiation Units and Measurements Report 89, target volumes and OARs were contoured [23]. The cumulative dose (EBRT + brachytherapy) to high-risk clinical target volume (HR-CTV) D_90_ was planned to be ≥85 Gy (EQD2). According to National Comprehensive Cancer Network guidelines [5], the cumulative dose constraints for OARs (D_2cc_) were: bladder ≤ 90 Gy, bowel ≤ 75 Gy, and rectum ≤ 75 Gy.

### 2.8. Cumulative Dose Calculation

The cumulative doses from EBRT and brachytherapy were converted into EQD2 using the linear-quadratic model. For EBRT, EQD2 was calculated on a fraction-by-fraction basis using the dose contribution from each adapted plan and then summed across all fractions. For each dose component, EQD2 was calculated according to the following equation: EQD2 = D × (d + α/β)/(2 + α/β), where D represents the total physical dose, d represents the dose per fraction, and the α/β ratio was assumed to be 10 Gy for tumor tissue (EQD2_10_) and 3 Gy for OARs (EQD2_3_).

The cumulative EQD2 was calculated by summing the fraction-level EBRT EQD2 contributions and the brachytherapy EQD2 contribution: EQD2_total_ = EQD2_EBRT_ + EQD2_BT_.

### 2.9. Assessment

The duration of each oART session was measured from the initiation of the first pre-treatment iCBCT to the completion of beam delivery. This interval included acquisition of the initial iCBCT, target and OAR contouring and editing, plan generation and selection, acquisition of the verification iCBCT, and delivery of the treatment beams. Dosimetric parameters for both the adapted and scheduled plans were extracted from the Ethos treatment planning system and analyzed using Python (version 3.10). Target coverage was evaluated using V_100%_, V_98%_, V_95%_, and V_90%_ for all CTVs and PTVs. OAR assessment included mean dose (D_mean_), D_2cc_, and volumetric dose metrics (V_10Gy_, V_20Gy_, V_30Gy_, V_40Gy_) for the bladder, rectum, and small bowel, along with monitoring of femoral head and bone marrow dose metrics. The dosimetric comparison was performed on a per-fraction basis, and no cumulative delivered-dose analysis across fractions was performed.

Acute toxicities were defined as those occurring within 90 days from the start of radiotherapy, according to the Common Terminology Criteria for Adverse Events version 5.0. Follow-up visits included physical examinations, complete blood count, comprehensive metabolic panel, tumor marker assessments, thoracic and abdominal CT scans, and pelvic CT or MRI scans. Tumor response was assessed according to the Response Evaluation Criteria in Solid Tumors version 1.1 using gynecologic examination and pelvic MRI, supplemented by CT to exclude distant disease. Complete clinical response was defined as the absence of residual disease on MRI together with no visible or palpable tumor on gynecologic examination. PET-CT was performed only when recurrence or metastatic disease was clinically suspected. All evaluations were performed locally by experienced radiation oncologists.

### 2.10. Sample Size

This prospective single-arm study was designed as an early-phase feasibility and safety study. Acute Grade ≥ 3 gastrointestinal toxicity was selected as the key safety endpoint for sample size justification. Using a one-sample exact binomial framework with a one-sided α of 0.05, 28 evaluable patients would provide more than 80% power to rule out an unacceptable acute Grade ≥ 3 gastrointestinal toxicity rate of 30%, assuming a true toxicity rate of 10%. Under this design, the predefined early safety criterion was considered met if the one-sided exact 95% upper confidence limit for the observed acute Grade ≥ 3 gastrointestinal toxicity rate was below 30%. Therefore, a target sample size of 30 patients was considered adequate for this early-phase feasibility and safety assessment.

### 2.11. Statistical Analysis

Data were analyzed using SPSS version 25.0 (IBM, Armonk, NY, USA). Continuous variables with normal distribution are presented as mean ± standard deviation, while non-normally distributed variables are presented as median (interquartile range). For the dosimetric comparison between adapted and scheduled plans, each dosimetric parameter was first summarized across the 17 treatment fractions for each patient separately for the two plan types. Patient-level summary values were then compared using paired *t*-tests or Wilcoxon signed-rank tests, as appropriate. Categorical variables are expressed as counts and percentages and compared using the chi-square test or Fisher’s exact test. All tests were two-sided, and *p* < 0.05 was considered statistically significant.

## 3. Results

### 3.1. Patient Characteristics

From September 2023 to April 2024, 39 patients were assessed for eligibility, of whom 30 were included in the final analysis. The median age was 57 years. According to FIGO 2018 staging, 43.3% of patients were classified as stage IIB and 20.0% as stage IIIC1. All patients with FIGO stage IIIC1 cervical cancer received a simultaneous integrated boost to metastatic lymph nodes during MHRT. Seventeen (56.7%) patients received five or more cycles of concurrent cisplatin. The median number of concurrent cisplatin cycles was 5 (range, 3–6; 3 patients received 3 cycles, 10 received 4 cycles, 15 received 5 cycles, and 2 received 6 cycles). Thirteen patients received fewer than five cycles, mainly because of treatment-related hematologic toxicity and reduced treatment tolerance. Detailed clinical characteristics of the enrolled patients are presented in Table 1. The median duration of EBRT was 22 days, while the median overall treatment time was 43 days. All patients successfully completed MRI- or CT-guided brachytherapy. Twenty-seven (90.0%) patients received intracavitary brachytherapy alone, whereas three (10.0%) patients required combined intracavitary/interstitial brachytherapy.

### 3.2. Workflow and Timing Data

All enrolled patients completed the planned 17 fractions of MHRT-oART, with 510 adaptive treatment fractions delivered in total. The first-attempt adaptation success rate was 99.0% per fraction, and adapted plans were selected in 99.4% of treatment fractions. Among the remaining three fractions, one was not selected due to inadequate bowel preparation, one because of suboptimal target coverage with the adapted plan, and one because of excessive OAR doses resulting from intrafractional anatomical changes.

The average online adaptive time, from the first iCBCT acquisition to plan selection, was 17 min and 20 s. The average total time per fraction was 23 min and 18 s. Detailed time distributions are shown in Table 2.

### 3.3. Target Dose Dosimetric Outcomes

**Figure 4:**
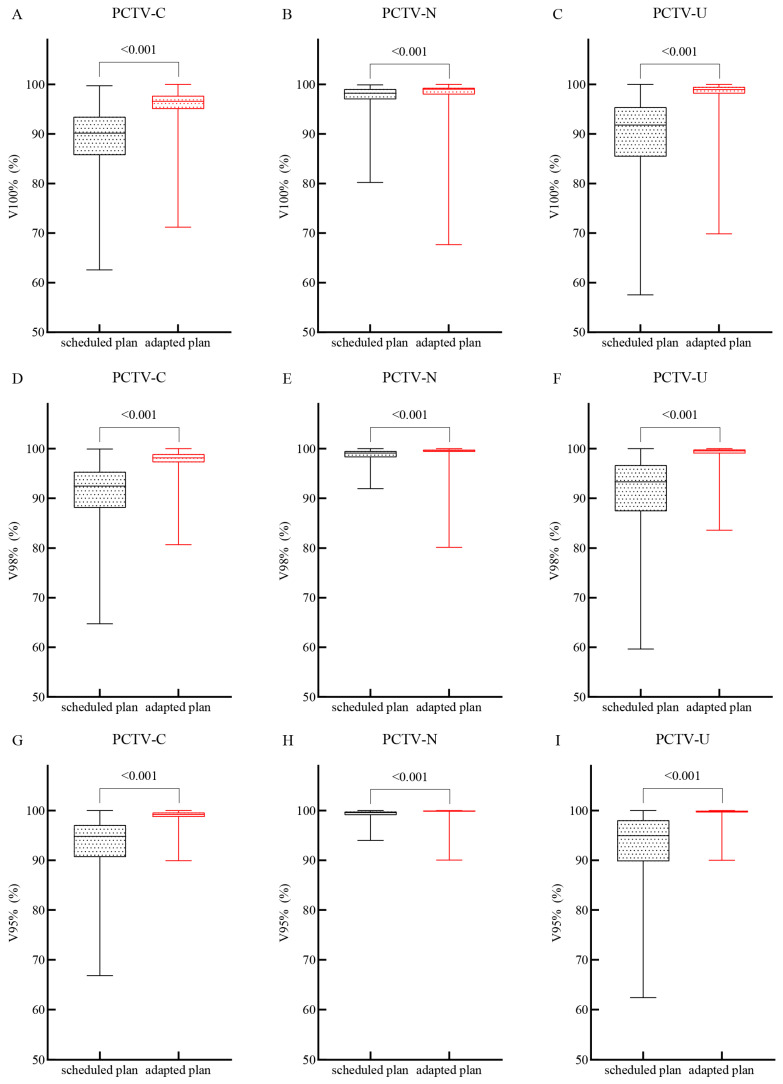
Distribution of V_100%_ (**A**–**C**), V_98%_ (**D**–**F**), and V_95%_ (**G**–**I**) coverage in scheduled and adapted plans. Panels (**A**,**D**,**G**) represent PCTV-C; (**B**,**E**,**H**) represent PCTV-N; and (**C**,**F**,**I**) represent PCTV-U. PCTV-U, planning target volume of the uterus; PCTV-C, planning target volume of the cervix; PCTV-N, planning target volume of the lymph nodes. For each patient, the median value across 17 treatment fractions was calculated separately for the two plan types, and the resulting paired patient-level values were compared using the Wilcoxon signed-rank test. The boxplots represent the distribution of patient-level median values across the 30 patients.

The comparison of dosimetric results between the adapted and scheduled plans is shown in Table 3, with the adapted plan demonstrating significantly improved target dose coverage. To evaluate plan quality, we employed the V_100%_ of the CTV as the primary criterion to represent target coverage for plan selection. As illustrated in Figure 4A–C, the adapted plans consistently showed significantly improved target coverage in V_100%_. Among the 510 fractions, the scheduled plans exhibited suboptimal V_100%_ coverage (below the required 95%) in 363 fractions for the planning target volume of the uterus (PCTV-U), compared to only 21 fractions in the adapted plans. All comparisons of V_100%_ showed statistically significant differences (all *p* < 0.001), with the most notable improvements observed in V_100%_ of PCTV-U (improved by 7.26%) and V_100%_ of the planning target volume of the cervix (PCTV-C) (improved by 8.79%).

All V_98%_ and V_95%_ comparisons demonstrated statistically significant differences (*p* < 0.001), as shown in Figure 4D–I. We further evaluated the proportion of fractions achieving V_95%_ ≥ 99% for each PTV. For PCTV-U and PCTV-C, the adapted plans exhibited suboptimal V_95%_ coverage (below 99%) in 35 and 109 fractions, respectively, whereas the scheduled plans showed substantially higher non-compliance rates of 444 and 486 fractions.

### 3.4. OAR Dosimetric Outcomes

**Figure 5:**
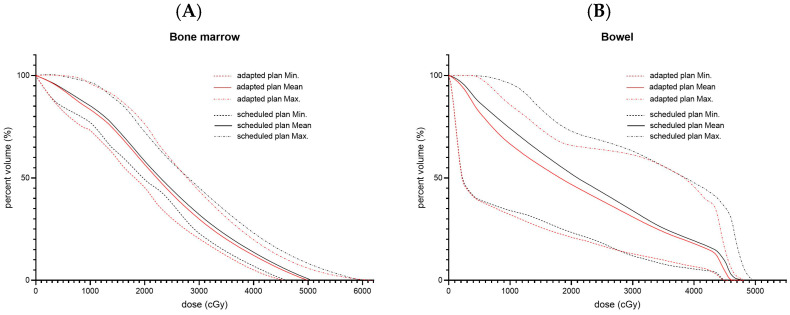

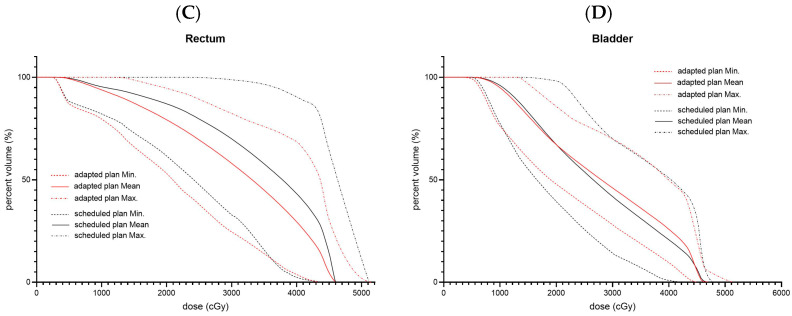
Dose–volume histograms comparing adapted and scheduled plans for bone marrow (**A**), bowel (**B**), rectum (**C**), and bladder (**D**).

Improved sparing of OARs was observed in the adapted plans compared to the scheduled plans, as shown in Table 4. Specifically, the adapted plans significantly reduced the V_10Gy_, V_20Gy_, V_30Gy_, V_40Gy_, and D_2cc_ for the rectum and small bowel. Significant reductions were also seen in the bladder V_10Gy_, V_20Gy_, V_40Gy,_ and D_2cc_ (all *p* < 0.001). In addition, the adapted plans resulted in notable dose reductions to the bone marrow and femoral heads. Dose–volume histograms illustrating these differences are presented in Figure 5.

### 3.5. Cumulative EQD2 Dose Assessment

The cumulative EQD2 doses combining EBRT and brachytherapy were evaluated for all patients. The median cumulative HR-CTV D90 (EQD2_10_) was 86.05 Gy (85.50, 87.65 Gy). The median cumulative OAR D2cc (EQD2_3_) values were 87.20 Gy (85.70, 89.50 Gy) for the bladder, 69.20 Gy (66.88, 72.24 Gy) for the rectum, and 71.33 Gy (66.92, 73.99 Gy) for the bowel. The cumulative doses to most OARs were within the recommended dose constraints.

### 3.6. Acute Toxicities

Acute toxicities are summarized in Table 5. Gastrointestinal toxicities occurred in 80% of patients and were primarily characterized by nausea, diarrhea, and abdominal discomfort. Nausea was reported in 70% of patients, predominantly Grade 1–2. Diarrhea occurred in 63.3% of patients, including three Grade 3 cases (10%), all of which resolved within one week with antidiarrheal therapy. Constipation (10%) and abdominal pain (30%) were generally mild, and the single case of intestinal obstruction (3.3%) was Grade 2 and resolved with conservative management. No Grade 4 or higher gastrointestinal events were observed. Genitourinary toxicities occurred in 40% of patients, mainly presenting as urinary frequency or urgency (26.7%) and dysuria (33.3%), almost all of which were Grade 1–2. No Grade ≥ 3 genitourinary toxicity was observed. Hematologic toxicity was common, affecting 90% of patients. Leukopenia was observed in 53.3% (36.7% Grade 3), neutropenia in 70% (10% Grade 3; 3.3% Grade 4), and anemia in 66.7% (one Grade 3 event). Thrombocytopenia occurred in 30% of patients and was limited to Grade 1–2. All hematologic toxicities were effectively managed with supportive care, and none resulted in treatment interruption.

Overall, most acute gastrointestinal and genitourinary toxicities were mild to moderate, whereas Grade ≥ 3 hematologic toxicity occurred in 40% of patients. Acute Grade 3 gastrointestinal toxicity occurred in 10% of patients, with a one-sided exact 95% upper confidence limit of 23.9%. Because this upper confidence limit was below the prespecified unacceptable rate of 30%, the predefined early safety criterion was met. Grade ≥ 3 hematologic toxicity occurred in 40% of patients, whereas no Grade ≥ 3 genitourinary toxicity or any Grade 4 nonhematologic events were observed. All 30 patients completed the three-month response assessment and were included in follow-up analyses. Tumor response was highly favorable, with all patients achieving a complete clinical response at the three-month post-treatment assessment.

## 4. Discussion

The present study should be interpreted as an early prospective feasibility study evaluating the integration of daily oART into a moderately hypofractionated definitive chemoradiotherapy program for cervical cancer, rather than the introduction of a novel radiotherapy platform. Although oART has previously been investigated in postoperative and definitive cervical cancer by our group and others, prospective evidence regarding its application to a moderately hypofractionated definitive regimen has remained limited. Therefore, the principal contribution of this study lies in demonstrating the feasibility, workflow efficiency, dosimetric benefits, acceptable acute toxicity, and encouraging early tumor response of this specific treatment strategy.

In this study, the workflow achieved a high per-fraction adaptation success rate (99.0%) and a mean total treatment duration of approximately 23 min, reflecting efficient implementation and streamlined clinical integration. These findings indicate that daily adaptation can be reliably incorporated into a hypofractionated regimen, where larger fraction sizes place greater demands on target coverage accuracy and OAR sparing. Importantly, treatment was well tolerated, with most acute toxicities being mild to moderate and manageable without treatment interruption. Beyond its technical feasibility, MHRT-oART demonstrated encouraging early tumor response, with a 100% complete clinical response rate at 3 months. However, in the absence of a conventional-fractionation control group, these favorable early outcomes cannot be attributed specifically to MHRT-oART, because the potential influences of patient selection, institutional expertise, and temporal trends cannot be excluded. Collectively, the favorable workflow efficiency, and acceptable toxicity profile observed in this study compare favorably with previously reported adaptive radiotherapy experiences in gynecologic malignancies and support the clinical feasibility of integrating daily oART into definitive treatment strategies for cervical cancer [16,18,19].

Beyond CBCT-based oART, MRI-guided adaptive radiotherapy (MRgART) has also emerged as a promising strategy for cervical cancer because of its superior soft-tissue visualization and capability for online adaptive treatment [24]. Early clinical experience has demonstrated the feasibility of MRgRT in patients with locally advanced cervical cancer. Boldrini et al. reported the clinical experience of low-field MRgRT in locally advanced cervical cancer, showing that MRgRT was feasible and safe, with favorable toxicity profiles and comparable treatment responses to standard linac-based radiotherapy [25]. Compared with CBCT-guided adaptation, MRgART enables more accurate visualization of the uterus, cervix, and surrounding pelvic organs, facilitating improved target delineation, individualized margin reduction, and adaptive replanning in response to substantial interfraction anatomical changes [24,26]. However, despite these technical advantages, MRgART remains limited by longer treatment times, higher equipment and operational costs, and limited worldwide availability, which currently restrict its widespread clinical implementation [26,27]. In contrast, CBCT-based oART has become increasingly accessible in routine clinical practice and provides a practical balance between treatment efficiency and adaptive precision. Therefore, our findings support CBCT-guided oART as a clinically feasible platform for implementing MHRT, while future prospective studies directly comparing CBCT- and MRI-guided adaptive workflows are warranted.

A key finding of this study is the substantial improvement in target dose coverage achieved by the adapted plans across all CTV and PTV subregions. Scheduled plans frequently failed to meet V_100%_ and V_95%_ objectives because of pronounced cervix–uterine interfractional motion, particularly affecting PCTV-U and PCTV-C. Daily oART effectively corrected these geometric uncertainties, increasing V_100%_ for PCTV-U and PCTV-C by 7.26% and 8.79%, respectively. The consistent improvements observed in V_98%_ and V_95%_ further demonstrate that oART enhances the robustness and accuracy of dose delivery—an advantage that becomes increasingly important in hypofractionated regimens, where fewer fractions reduce opportunities for mid-course adjustment.

Beyond improved target coverage, MHRT-oART also achieved meaningful dose reductions across multiple OARs, including the bladder, rectum, bowel, bone marrow, and femoral heads. Notably, decreases in rectal and small bowel V_30Gy_ and V_40Gy_ are clinically relevant, as these dose–volume metrics are closely linked to acute gastrointestinal toxicity and the risk of treatment interruptions during definitive chemoradiotherapy [13]. Adaptation resulted in lower D_mean_ values for all major pelvic organs, consistent with prior adaptive workflows in gynecologic cancers demonstrating that margin reduction and real-time anatomic correction effectively decrease OAR dose. Given that larger per-fraction doses in MHRT may theoretically heighten mucosal sensitivity, these dose reductions are particularly relevant for mitigating acute toxicity risk [13,18]. The ability of oART to limit unnecessary OAR exposure therefore provides a strong rationale for its integration into hypofractionated pelvic irradiation. However, as the scheduled plan was an internal Ethos comparator and no cumulative dose accumulation was performed, the observed differences should be interpreted as per-fraction dosimetric benefits of online adaptation. Because scheduled and adapted plans are available only for the EBRT component, cumulative dosimetric comparisons between the two strategies are not feasible. Nevertheless, cumulative EBRT plus brachytherapy EQD2 values remained within accepted dose constraints for all major OARs, providing additional context for the favorable acute toxicity profile observed in this prospective cohort.

The choice of fractionation schedule also warrants consideration. This schedule was selected with reference to previously reported hypofractionated cervical cancer regimens. A randomized study using 40 Gy in 15 fractions with concurrent cisplatin and brachytherapy reported a higher rate of acute Grade ≥ 3 gastrointestinal toxicity than conventional chemoradiotherapy, whereas the HYPOCx-iRex trial evaluated a more conservative regimen of 44 Gy in 20 fractions. Compared with these regimens, 43.35 Gy in 17 fractions represents an intermediate approach that maintains a conventional-like tumor EQD2 while reducing treatment duration [13,15]. The use of daily oART was intended to mitigate the potential toxicity risk associated with hypofractionation by accounting for interfractional anatomical changes before each fraction.

In terms of clinical safety, the acute gastrointestinal and genitourinary toxicities observed in this study were acceptable and consistent with historical reports of definitive chemoradiotherapy. For example, in a clinical cohort of 50 patients treated with IMRT plus weekly cisplatin, 46% experienced acute Grade ≥ 2 gastrointestinal toxicity [28]. Similarly, in a large retrospective IMRT cohort of FIGO IIIC1 cervical cancer patients, acute proctitis occurred in 12.8% (Grade 1), 4.5% (Grade 2), and 0.6% (Grade 3), while frequent urination was reported in 24.2% (Grade 1) and 16.1% (Grade 2) [29]. In contrast, no acute proctitis occurred in our study, and Grade 1–2 urinary frequency was limited to 13.3% each, with no Grade ≥ 3 genitourinary events. Direct comparisons with historical conventional IMRT cohorts remain limited by differences in patient characteristics, treatment regimens, toxicity assessment, and study design. Compared with other MHRT studies, the rate of Grade ≥ 3 acute gastrointestinal toxicity in our cohort (10%) was lower than that reported in the HYPOCx-iRex trial (40%) and similar to rates from a resource-limited hypofractionation study [15,30]. Nevertheless, the observed reductions in rectal and bowel dose were not accompanied by an obviously low acute gastrointestinal toxicity rate in this cohort. This apparent discrepancy may reflect several factors. The adapted-versus-scheduled comparison was a per-fraction planning analysis rather than a comparison of cumulative delivered dose, and acute gastrointestinal toxicity is also influenced by concurrent chemotherapy, patient-related factors, bowel preparation and motion, and brachytherapy. The acceptable gastrointestinal and genitourinary toxicity profile observed in this study may also be associated with controlled cumulative radiation exposure. In our cohort, cumulative EQD2 values of the bladder, rectum, bowel, and sigmoid colon remained within recommended dose constraints after integrating EBRT and brachytherapy, supporting the feasibility of this MHRT-oART regimen. In addition, the higher dose per fraction used in MHRT may increase the biological effect on early-responding gastrointestinal mucosa and may partly offset the potential benefit of improved OAR sparing [13,15]. No Grade ≥ 3 genitourinary toxicity occurred, whereas Grade ≥ 3 hematologic toxicity occurred in 40% of patients. This likely reflected the combined effects of concurrent cisplatin and pelvic bone marrow irradiation. Although adapted plans significantly reduced bone marrow V_10Gy_, V_20Gy_, and D_90_, the absolute reductions were modest, and neither functional bone marrow imaging nor prospectively defined bone marrow-sparing constraints were incorporated into the treatment protocol. Previous studies have demonstrated an association between pelvic bone marrow dose and acute hematologic toxicity and suggested that dedicated bone marrow-sparing strategies may reduce this risk [31,32]. Therefore, the observed dosimetric improvements may contribute to toxicity mitigation, although their clinical impact requires further evaluation.

Our findings provide clinically meaningful support for the potential role of MHRT in cervical cancer. Prolonged overall treatment time with conventional 1.8–2.0 Gy fractionation remains a major limitation, as multiple studies have shown that extended treatment duration is strongly associated with poorer local control and survival [33,34,35,36,37] MHRT offers a strategy to shorten treatment, improve efficiency, and reduce resource burden without compromising therapeutic outcomes. Although current evidence for definitive MHRT remains limited and heterogeneous, our results show that when combined with daily oART, MHRT can be delivered safely and with high dosimetric quality. These observations align with emerging postoperative MHRT data, such as the POHIM_RT and POHIM_CCRT trials, which demonstrated that 40 Gy in 16 fractions can reduce treatment duration while maintaining low severe toxicity rates [38,39], and extend these findings to the definitive chemoradiotherapy setting, where target motion is more pronounced.

Another important consideration is the generalizability of the present findings. As an early-phase prospective feasibility and safety study, this trial intentionally enrolled a relatively selected population with FIGO 2018 stage IB1–IIB or IIIC1 disease, limited pelvic nodal burden, no common iliac lymph node involvement, and good performance status. These eligibility criteria were chosen to minimize clinical heterogeneity and facilitate the initial evaluation of the feasibility, workflow efficiency, dosimetric benefits, and early safety of integrating daily oART into an MHRT regimen, consistent with previous prospective studies of hypofractionated chemoradiotherapy for cervical cancer and early clinical implementation of oART [13,15,40]. Consequently, the present findings should be interpreted primarily for carefully selected patients and should not be directly extrapolated to those with bulky pelvic nodal disease, common iliac or para-aortic lymph node involvement, more advanced disease, or poorer performance status, who generally require larger treatment volumes and more complex radiotherapy planning [3]. Based on the current evidence, daily oART-guided MHRT appears most suitable for carefully selected patients with limited pelvic nodal disease undergoing definitive chemoradiotherapy, whereas conventional fractionation remains the preferred approach for patients with more extensive disease until additional prospective evidence becomes available. Future multicenter studies including broader patient populations are warranted to further establish the efficacy, safety, and generalizability of this treatment strategy. Our ongoing multicenter phase III SWIFT-1 trial (NCT06641635), which adopts broader eligibility criteria, is expected to provide further evidence in a more representative population [22].

Although daily oART was feasible in this study, routine implementation requires additional personnel and treatment resources. Each fraction involved physician review of target and OAR contours, plan evaluation, and verification imaging, with at least one radiation oncologist and two radiation therapists participating and physicist support available when necessary. The associated personnel demands, machine occupancy, training requirements, and need for adaptive-capable equipment may limit broader implementation, particularly in resource-constrained centers [41,42]. The mean treatment time of 23 min and 18 s also allowed the possibility of intrafraction anatomical changes. A second iCBCT was therefore acquired before treatment delivery, and clinically relevant changes prompted repeat adaptation. Our previous postoperative gynecologic oART study showed that, with rigorous bladder and rectal preparation, intrafraction variations over a similar interval had limited dosimetric impact [43]. Nevertheless, the intact uterus and cervix in the definitive setting may exhibit greater mobility, and further evaluation using systematic motion assessment and delivered-dose accumulation is warranted.

This study has several limitations, including the relatively small sample size, limited follow-up, and single-arm design. The current follow-up supports assessment of feasibility and acute toxicity and is insufficient to comprehensively evaluate late radiation-related toxicity or long-term oncologic outcomes. In addition, the reliance on an oART platform may limit generalizability to centers without adaptive capabilities. Furthermore, because this was an early-phase feasibility study with relatively strict eligibility criteria, the present findings are primarily applicable to carefully selected patients with limited nodal burden and squamous histology. Broader applicability should be evaluated in future randomized studies with expanded eligibility criteria. Nevertheless, the high adaptation success rate and efficient workflow observed in this study support the feasibility of moderately hypofractionated oART in routine clinical practice. Importantly, adaptive radiotherapy is increasingly recognized as a major direction in the evolution of modern radiation oncology [44]. With leading vendors actively advancing adaptive platforms through improvements in automation, artificial intelligence-assisted contouring, and treatment planning, broader clinical adoption is anticipated in the coming years.

Future prospective randomized studies with larger cohorts and longer follow-up are warranted to further define the efficacy, safety, and optimal implementation of moderately hypofractionated oART for cervical cancer, and to identify the clinical and anatomical characteristics of patients most likely to benefit from daily online adaptation.

## 5. Conclusions

This prospective study provides early evidence supporting the feasibility of integrating daily oART into a moderately hypofractionated definitive chemoradiotherapy program for cervical cancer. Daily oART-guided MHRT demonstrated an efficient clinical workflow, improved target coverage and OAR sparing compared with scheduled plans, and acceptable acute toxicity. Given the prospective single-arm design, relatively small sample size, and limited follow-up, these findings should be interpreted as preliminary evidence supporting the feasibility of this combined treatment strategy. Further validation in larger multicenter prospective studies with longer follow-up is warranted before broader clinical adoption.

## Figures and Tables

**Table 1:** Clinicopathologic characteristics.

Characteristics	Number (n)	Percent (%)
Age (years)		
Median	57	
<60	19	63.3
≥60	11	36.7
Tumor size		
<4 cm	19	63.3
≥4 cm	11	36.7
SCC Ag at diagnosis		
≤1.5 ng/mL	7	23.3
>1.5 ng/mL	23	76.7
FIGO 2018 stage		
IB1	2	6.7
IB2	4	13.3
IIA1	4	13.3
IIA2	1	3.3
IIB	13	43.3
IIIC1	6	20.0
Chemotherapy cycles		
<5 cycles	13	43.3
≥5 cycles	17	56.7

FIGO, International Federation of Gynecology and Obstetrics; SCC Ag, squamous cell carcinoma antigen.

**Table 2:** Treatment workflow timing for moderately hypofractionated online adaptive radiotherapy.

Time (Minutes and Seconds)	Average	Min	Max
First iCBCT acquisition	52 s	40 s	59 s
CTV and OARs creation and adjustment	12 min 26 s	3 min 2 s	29 min 17 s
Plan creation and selection	4 min 6 s	3 min 2 s	10 min 13 s
Second iCBCT acquisition	50 s	41 s	60 s
Beam-on time	4 min 30 s	3 min 30 s	6 min 43 s
Online adaptive time	17 min 20 s	7 min 38 s	42 min 36 s
Treatment delivery time	5 min 22 s	4 min 13 s	7 min 37 s
Total time	23 min 18 s	15 min 4 s	47 min 57 s

iCBCT, iterative cone-beam computed tomography; CTV, clinical target volume; OARs, organ at risk. Online adaptive time was defined as the duration from the first iCBCT acquisition to the plan selection; the treatment delivery time referred to the period from the second iCBCT acquisition to the completion of beam delivery; the total treatment time was defined as the interval from the first iCBCT acquisition to the end of beam delivery.

**Table 3:** Comparison of dosimetric results for targets between adapted plans and scheduled plans during online adaptive external beam radiotherapy.

Targets	Dosimetric Parameter	Adapted Plan	Scheduled Plan	*p* Value
CTV-N	V_100%_ (%)	99.96 (99.81, 99.99)	99.95 (99.83, 99.99)	0.021
	V_98%_ (%)	100 (100, 100)	99.99 (99.95, 100)	<0.001
	V_95%_ (%)	100 (100, 100)	100 (99.99, 100)	<0.001
	V_90%_ (%)	100 (100, 100)	100 (100, 100)	<0.001
CTV-U	V_100%_ (%)	100 (100, 100)	97.96 (90.51, 99.83)	<0.001
	V_98%_ (%)	100 (100, 100)	98.87 (92.40, 100)	<0.001
	V_95%_ (%)	100 (100, 100)	99.49 (94.33, 100)	<0.001
	V_90%_ (%)	100 (100, 100)	99.92 (96.72, 100)	<0.001
CTV-C	V_100%_ (%)	99.94 (99.75, 100)	96.86 (92.04, 98.97)	<0.001
	V_98%_ (%)	100 (99.97, 100)	97.95 (93.89, 99.52)	<0.001
	V_95%_ (%)	100 (98.99, 100)	95.54 (64.67, 98.89)	<0.001
	V_90%_ (%)	100 (99.78, 100)	97.14 (68.13, 99.70)	<0.001
PCTV-N	V_100%_ (%)	99.03 (97.94, 99.31)	98.22 (96.95, 99.04)	<0.001
	V_98%_ (%)	99.64 (99.33, 99.79)	99.10 (98.23, 99.51)	<0.001
	V_95%_ (%)	99.90 (99.83, 99.96)	99.58 (99.08, 99.81)	<0.001
	V_90%_ (%)	99.99 (99.98, 100)	99.88 (99.66, 99.97)	<0.001
PCTV-U	V_100%_ (%)	98.94 (98.10, 99.49)	91.77 (85.40, 95.39)	<0.001
	V_98%_ (%)	99.55 (99.03, 99.82)	93.32 (87.39, 96.69)	<0.001
	V_95%_ (%)	99.88 (99.61, 99.97)	94.93 (89.79, 98.04)	<0.001
	V_90%_ (%)	100 (99.93, 100)	96.93 (92.56, 99.16)	<0.001
PCTV-C	V_100%_ (%)	96.59 (95.58, 97.70)	90.15 (85.70, 93.46)	<0.001
	V_98%_ (%)	98.14 (97.25, 98.90)	92.47 (88.06, 95.34)	<0.001
	V_95%_ (%)	99.41 (99.05, 99.67)	94.80 (90.62, 97.09)	<0.001
	V_90%_ (%)	99.86 (99.67, 99.97)	97.13 (93.57, 98.66)	<0.001

OARs, organs at risk; CTV-N, clinical target volume of the lymph nodes; CTV-U, clinical target volume of uterus; CTV-C, clinical target volume of cervix; PCTV-N, planning target volume of the lymph nodes; PCTV-U, planning target volume of the uterus; PCTV-C, planning target volume of the cervix. Values are presented based on patient-level summaries across 30 patients. *p* values were obtained using paired Wilcoxon signed-rank tests.

**Table 4:** Comparison of dosimetric results for organs at risk between adapted plans and scheduled plans during online adaptive external beam radiotherapy.

Organs at Risk	Dosimetric Parameter	Adapted Plan	Scheduled Plan	*p* Value
Bladder	D_mean_ (cGy)	2749 ± 330.8	2822 ± 263.7	<0.001
	V_10Gy_ (%)	97.10 (91.41, 99.25)	99.39 (92.89, 100)	<0.001
	V_20Gy_ (%)	66.76 (59.64, 75.41)	68.38 (59.44, 74.59)	<0.001
	V_30Gy_ (%)	42 (34.29, 49.14)	46.50 (39.96, 52.23)	0.532
	V_40Gy_ (%)	20.14 (13.29, 27.31)	25.40 (20.94, 31.18)	<0.001
	D_2cc_ (cGy)	4548 (4522, 4578)	4573 (4520, 4611)	<0.001
Rectum	D_mean_ (cGy)	3090 ± 264.6	3443 ± 332.6	<0.001
	V_10Gy_ (%)	94.56 (90.72, 97.35)	96.04 (92.79, 98.77)	<0.001
	V_20Gy_ (%)	80.17 (74.74, 84.84)	87.81 (82.60, 92.93)	<0.001
	V_30Gy_ (%)	58.53 (50.91, 64.92)	70.58 (61.51, 78.99)	<0.001
	V_40Gy_ (%)	29.24 (21.82, 36.72)	41.72 (32.96, 52.39)	<0.001
	D_2cc_ (cGy)	4490 (4426, 4522)	4546 (4512, 4581)	<0.001
Bowel	D_mean_ (cGy)	2095 ± 267.4	2281 ± 318.1	<0.001
	V_10Gy_ (%)	66.91 (61.56, 73.31)	74.72 (68.89, 81.14)	<0.001
	V_20Gy_ (%)	47.58 (42.72, 51.27)	52.61 (47.77, 57.36)	<0.001
	V_30Gy_ (%)	30.83 (24.82, 36.92)	34.29 (26.36, 41.02)	<0.001
	V_40Gy_ (%)	16.84 (13.15, 21.36)	18.37 (13.29, 23.70)	<0.001
	D_2cc_ (cGy)	4580 (4553, 4615)	4646 (4610, 4746)	<0.001
	D_5cc_ (cGy)	4555 (4536, 4574)	4613 (4583, 4677)	<0.001
Bone marrow	V_10Gy_ (%)	82.23 (79.18, 86.20)	84.24 (81.36, 88.35)	<0.001
	V_20Gy_ (%)	55.50 (53.32, 58.37)	56.68 (54.87, 60.84)	<0.001
	D_90%_ (cGy)	599 (494.5, 779.5)	688 (505.5, 886.5)	<0.001
Left femur head	D_mean_ (cGy)	1396 (1338, 1467)	1416 (1357, 1487)	<0.001
	D_5%_ (cGy)	2460 (2304, 2624)	2585 (2344, 2808)	<0.001
	V_30Gy_ (%)	1.32 (0.47, 2.40)	2.28 (0.65, 3.60)	<0.001
Right femur head	D_mean_ (cGy)	1388 (1318, 1448)	1392 (1326, 1477)	0.001
	D_5%_ (cGy)	2451 (2296, 2657)	2459 (2252, 2826)	<0.001
	V_30Gy_ (%)	1.34 (0.52, 2.65)	1.68 (0.45, 3.91)	<0.001

Values are presented based on patient-level summaries across 30 patients. *p* values were obtained using paired *t*-tests or Wilcoxon signed-rank tests, as appropriate.

**Table 5:** Acute toxicity in patients receiving moderately hypofractionated online adaptive radiotherapy for cervical cancer.

Toxicities	Grade 0 (N, %)	Grade 1 (N, %)	Grade 2 (N, %)	Grade 3 (N, %)	Grade 4 (N, %)
Total	1 (3.3%)	1 (3.3%)	16 (53.3%)	11 (36.7%)	1 (3.3%)
Hematologic toxicity	3 (10%)	2 (6.7%)	13 (43.3%)	11 (36.7%)	1 (3.3%)
Leukopenia	5 (16.7%)	1 (3.3%)	13 (43.3%)	11 (36.7%)	0 (0%)
Neutropenia	8 (26.7%)	9 (30%)	9 (30%)	3 (10%)	1 (3.3%)
Anemia	10 (33.3%)	7 (23.3%)	12 (40%)	1 (3.3%)	0 (0%)
Thrombocytopenia	21 (70%)	5 (16.7%)	4 (13.3%)	0 (0%)	0 (0%)
Gastrointestinal toxicity	6 (20%)	6 (20%)	15 (50%)	3 (10%)	0 (0%)
Nausea	9 (30%)	7 (23.3%)	14 (46.7%)	0 (0%)	0 (0%)
Vomiting	18 (60%)	0 (0%)	12 (40%)	0 (0%)	0 (0%)
Constipation	27 (90%)	3 (10%)	0 (0%)	0 (0%)	0 (0%)
Diarrhea	6 (20%)	10 (33.3%)	11 (36.7%)	3 (10%)	0 (0%)
Abdominal pain	21 (70%)	4 (13.3%)	5 (16.7%)	0 (0%)	0 (0%)
Intestinal obstruction	29 (96.7%)	0 (0%)	1 (3.3%)	0 (0%)	0 (0%)
Genitourinary toxicity	18 (60%)	3 (10%)	9 (30%)	0 (0%)	0 (0%)
Urinary frequency/urgency	22 (73.3%)	4 (13.3%)	4 (13.3%)	0 (0%)	0 (0%)
Dysuria	20 (66.7%)	1 (3.3%)	9 (30%)	0 (0%)	0 (0%)
Cystitis	29 (96.7%)	0 (0%)	1 (3.3%)	0 (0%)	0 (0%)

The “Total” row represents the highest acute toxicity grade experienced by each patient across all toxicity categories.

## Data Availability

The data supporting this study can be obtained from the corresponding authors upon reasonable request.

## Abbreviations

The following abbreviations are used in this manuscript:

CBCT	Cone-beam computed tomography
CT	Computed tomography
CTV	Clinical target volume
CTV-C	Clinical target volume of the cervix
CTV-N	Clinical target volume of the lymph nodes
CTV-U	Clinical target volume of the uterus
EBRT	External beam radiotherapy
EQD2	Equivalent dose in 2-Gy fractions
FIGO	International Federation of Gynecology and Obstetrics
HR-CTV	high-risk clinical target volume
iCBCT	Iterative cone beam computed tomography
IMRT	Intensity-modulated radiation therapy
MHRT	Moderately hypofractionated radiotherapy
MRI	Magnetic resonance imaging
MRgART	MRI-guided adaptive radiotherapy
OARs	Organs at risk
oART	Online adaptive radiotherapy
PET-CT	Positron emission tomography-computed tomography
PTV	Planning target volume
PCTV-C	Planning target volume of the cervix
PCTV-N	Planning target volume of the lymph nodes
PCTV-U	Planning target volume of the uterus
